# Concurrent Long Thoracic and Lateral Cutaneous Intercostal Nerve Entrapment After Chest Tube Thoracostomy Causing Scapular Winging and Chest Wall Pain: Diagnosis With Ultrasound and Treatment With Hydrodissection

**DOI:** 10.1111/papr.70195

**Published:** 2026-09-11

**Authors:** Yonghyun Yoon, King Hei Stanley Lam, Daniel Chiung‐Jui Su, Teinny Suryadi, Anwar Suhaimi

**Affiliations:** ^1^ Hallym University College of Medicine Chuncheon‐si Republic of Korea; ^2^ Incheon Terminal Orthopedic Surgery Clinic Incheon Republic of Korea; ^3^ International Academy of Regenerative Medicine Incheon Republic of Korea; ^4^ International Association of Musculoskeletal Medicine Kowloon Hong Kong SAR China; ^5^ The University of Hong Kong Hong Kong Hong Kong SAR China; ^6^ The Chinese University of Hong Kong Hong Kong Hong Kong SAR China; ^7^ The Hong Kong Institute of Musculoskeletal Medicine Kowloon Hong Kong SAR China; ^8^ Chi Mei Hospital Chiali Tainan City Taiwan; ^9^ A Tempo Regeneration Center for Musicians Tainan Taiwan; ^10^ Department of Physical Medicine and Rehabilitation Hermina Podomoro Hospital North Jakarta Indonesia; ^11^ Department of Physical Medicine and Rehabilitation Medistra Hospital South Jakarta Indonesia; ^12^ Physical Medicine and Rehabilitation Synergy Clinic West Jakarta Indonesia; ^13^ Federal Territory of Kuala Lumpur Universiti Malaya Faculty of Medicine Kuala Lumpur Malaysia

**Keywords:** chest tube thoracostomy, hydrodissection, intercostal nerve, long thoracic nerve, musculoskeletal ultrasonography, neuropathic pain, scapular winging

## Abstract

**Background:**

Chest tube thoracostomy may cause iatrogenic nerve injury, resulting in persistent pain and functional impairment. Long thoracic nerve (LTN) palsy after chest tube placement has been described, but concurrent involvement of the LTN and the lateral cutaneous branch of an intercostal nerve (LCIN) appears to be rarely reported. We describe a patient with persistent post‐thoracostomy chest wall pain and scapular winging in whom musculoskeletal ultrasonography identified entrapment of both nerves.

**Case Presentation:**

A 43‐year‐old man presented with severe left shoulder and chest wall pain 6 months after chest tube placement for traumatic hemopneumothorax. Examination demonstrated focal tenderness, dysesthesia, and mechanical allodynia at the chest tube scar, along with medial/inferior scapular winging during serratus anterior provocative testing. Shoulder range of motion was full on examination; however, forward flexion beyond 15° markedly aggravated pain, resulting in pain‐related reluctance to move the shoulder. Manual muscle testing was 4+/5 before treatment because pain limited force generation. Musculoskeletal ultrasound demonstrated focal abnormality of the LTN where it pierced the axillary fascia over the sixth rib, with loss of fascicular definition and reduced glide, and tethering/thickening of the LCIN within scar tissue at the prior tube insertion site. Symptom reproduction with sonopalpation supported the clinical relevance of both findings.

**Intervention and Results:**

Ultrasound‐guided hydrodissection using 5% dextrose in water was performed around both nerves. A total of approximately 40 mL of 5% dextrose in water was used, with 20 mL administered per nerve. Immediately after the first procedure, pain improved from NRS 9/10 to 2/10, with marked reduction in pain during shoulder movement, including forward flexion. The patient subsequently underwent 5 treatment sessions at 2‐week intervals over 10 weeks. During follow‐up, manual muscle testing improved to 5/5 without pain, scapular winging was no longer evident on provocative testing, and the Shoulder Pain and Disability Index improved from pain/disability scores of 25/15 to 6/4. At the sixth follow‐up visit, pain and function were satisfactory, and the patient elected continued monitoring without further intervention. At 1‐year follow‐up, treatment efficacy remained sustained without recurrence.

**Conclusion:**

In patients with persistent shoulder or chest wall pain after chest tube thoracostomy, iatrogenic peripheral nerve entrapment should be considered. This case highlights the value of a focused neuromuscular examination and musculoskeletal ultrasound for identifying concurrent motor and sensory nerve involvement and suggests that ultrasound‐guided hydrodissection may be a useful minimally invasive treatment. Because electrodiagnostic studies were not performed, the findings are most consistent with entrapment/neurapraxia with dynamic tethering rather than electrodiagnostically proven axonal palsy.

## Executive Summary

Some patients continue to have shoulder or chest wall pain after a chest tube is inserted. In uncommon cases, this may occur because nearby nerves become irritated or trapped in scar tissue. This report describes a man who developed severe pain and shoulder blade “winging” after chest tube placement following a motorcycle accident.

Ultrasound examination suggested involvement of two nerves: the long thoracic nerve, which helps control the muscle that stabilizes the shoulder blade, and a lateral cutaneous branch of an intercostal nerve, which supplies sensation to the chest wall. Both nerves appeared to be affected near the previous chest tube site.

The patient was treated with ultrasound‐guided hydrodissection, a minimally invasive procedure in which a sugar‐water solution is injected around the nerves to separate them from surrounding scar tissue and soft tissue adhesions. His pain improved immediately after the first treatment and continued to improve over several sessions. Shoulder blade winging resolved, muscle strength improved, and the benefit was maintained at 1‐year follow‐up.

This case highlights peripheral nerve entrapment as an important possible cause of persistent pain and scapular dysfunction after chest tube thoracostomy. It also suggests that ultrasound‐guided hydrodissection may be a useful non‐surgical treatment option in selected patients.

## 1 Introduction

Chest tube thoracostomy is a common and often life‐saving procedure used to evacuate air, blood, or fluid from the pleural space [1,2]. Although generally safe, it may be associated with important complications, including infection, bleeding, malposition, injury to intrathoracic or abdominal organs, and peripheral nerve injury [1, 2, 3]. Despite its ubiquity, chest tube practice remains variable in several technical and management aspects, and parts of routine care continue to be guided more by tradition than by high‐quality evidence [4]. Among the less emphasized complications, iatrogenic neuropathy is clinically significant because it may lead to persistent pain, movement‐related disability, and delayed functional recovery.

The long thoracic nerve (LTN) is particularly relevant in this setting because of its vulnerable course along the lateral chest wall. Anatomical work has shown that the distal LTN commonly lies near the midaxillary line, especially around the fourth to sixth intercostal spaces and fifth to sixth rib levels, placing it at risk during lateral thoracic procedures [5]. In parallel, the lateral cutaneous branch of the intercostal nerve (LCIN) traverses the lateral thoracic fascia near the midaxillary line to supply sensation to the lateral chest wall [6]. The expected relationship of these structures to the prior chest tube scar in this case is illustrated schematically in Figure **1**. Injury or entrapment of the LTN may lead to serratus anterior dysfunction, medial scapular winging, shoulder pain, and impaired overhead function [6,7], whereas injury to the LCIN may produce focal neuropathic chest wall pain, dysesthesia, and allodynia [8,9].

**FIGURE 1:**
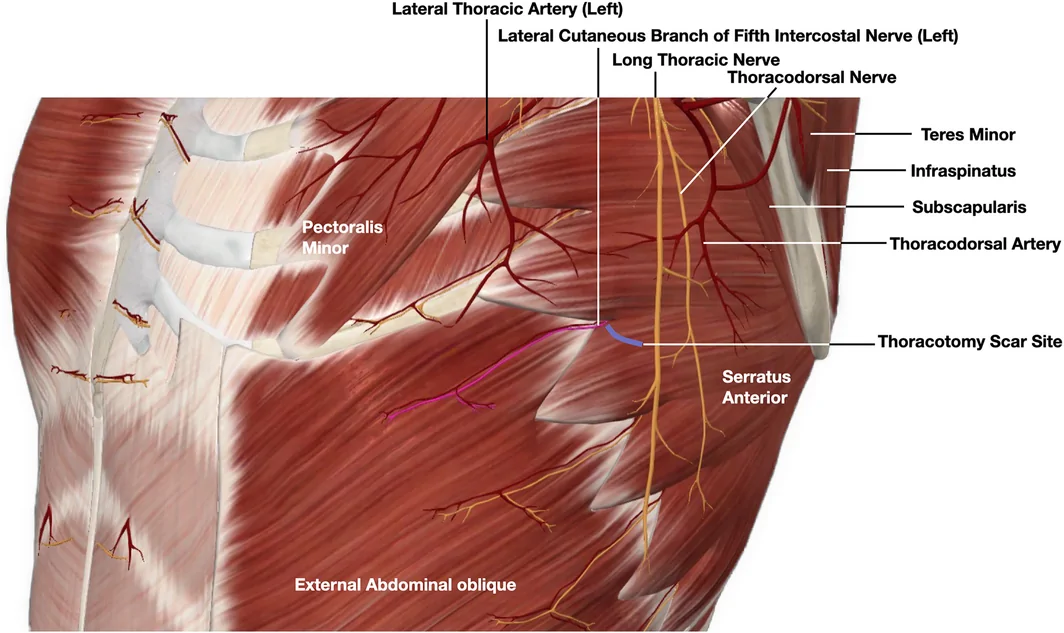
**Schematic anatomical illustration of the implicated nerves and prior chest tube scar**. The long thoracic nerve (LTN) courses along the superficial surface of the serratus anterior muscle. The lateral cutaneous branch of the intercostal nerve (LCIN) traverses the lateral thoracic fascia near the midaxillary line. The approximate location of the prior chest tube scar is shown in relation to these structures, illustrating the anatomical basis for concurrent motor and sensory nerve entrapment in this case.

Chest tube‐related scapular winging due to presumed LTN injury has been reported previously, but remains uncommon in the literature [7]. By contrast, sensory involvement of the LCIN in this setting has received little attention. Intercostal nerve impairment related to chest tube insertion has been objectively demonstrated and is a plausible contributor to persistent post‐procedural chest wall pain [8].

High‐resolution musculoskeletal ultrasonography (US) is well suited to this problem because it permits dynamic, side‐to‐side comparison of peripheral nerves, scar tissue, and fascial planes, while also allowing symptom provocation with sonopalpation and precise image‐guided intervention [9]. Ultrasound‐guided hydrodissection (HD), in which fluid is injected to separate a nerve from surrounding adhesions or constricting fascia, has emerged as a minimally invasive treatment for selected entrapment neuropathies [9].

We report a case of persistent shoulder and chest wall pain with scapular winging after chest tube thoracostomy in which ultrasound findings supported concurrent entrapment of the LTN and LCIN. This report aims to: (1) describe the clinical and sonographic features of this combined presentation, (2) discuss the relevant anatomy and likely mechanism of injury, and (3) illustrate the potential therapeutic role of ultrasound‐guided hydrodissection.

## 2 Case Report

A 43‐year‐old man presented to our physical medicine and rehabilitation clinic with a 6‐month history of severe left‐sided shoulder and chest wall pain following chest tube placement at an outside hospital after a motorcycle accident. His medical history included hypertension, diabetes mellitus, and hyperlipidemia. The original trauma had resulted in bilateral first‐rib fractures, an occipital bone fracture, and left second‐ through sixth‐rib fractures with hemopneumothorax. He underwent open reduction and internal fixation of the occipital fracture and tube thoracostomy for the thoracic injury.

### 2.1 Symptoms and Radiographs

At presentation, radiographs demonstrated bony union of the rib fractures. Despite fracture healing, he continued to experience severe left shoulder and lateral chest wall pain, rated 9/10 on the Numerical Rating Scale (NRS). He localized the most intense pain to the chest tube scar and adjacent lateral chest wall.

Physical examination revealed a hypertrophic scar at the prior chest tube insertion site with focal tenderness, dysesthesia, and mechanical allodynia. The area of maximal abnormal sensation is shown in Figure **2**A. Inspection demonstrated medial and inferior scapular winging during provocative maneuvers, including punch‐out and push‐up testing (Figure **2**B,C). These bedside tests supported abnormal scapulothoracic mechanics but were not considered specific, as wall push‐up‐type maneuvers may produce false‐positive winging patterns in patients without true serratus anterior dysfunction [10]. Shoulder range of motion was full on examination both before and after treatment. However, before treatment, forward flexion beyond 15° markedly aggravated pain, resulting in pain‐related reluctance to move the shoulder. Manual muscle testing demonstrated 4+/5 strength before treatment because pain limited force generation. At baseline, the Shoulder Pain and Disability Index (SPADI) showed a pain subscale score of 25 and a disability subscale score of 15.

**FIGURE 2:**
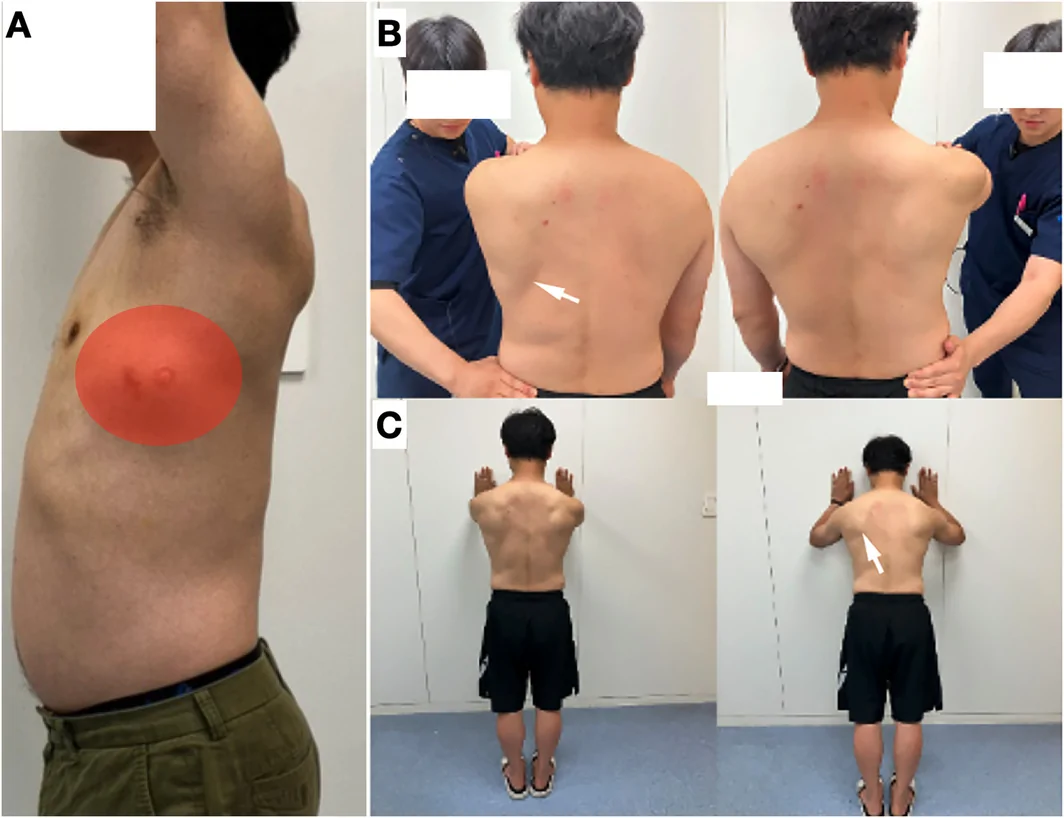
Clinical findings. (A) Body‐surface markings showing the area of maximal dysesthesia and mechanical allodynia surrounding the prior left chest tube scar. (B) Punch‐out maneuver demonstrating medial scapular winging (arrow). (C) Push‐up maneuver demonstrating increased medial/inferior winging of the left scapula (arrow).

### 2.2 Differential Diagnosis

Because scapular winging and chest wall pain may arise from several causes, the differential diagnosis included cervical radiculopathy, brachial plexopathy, spinal accessory neuropathy, dorsal scapular neuropathy, rotator cuff‐related shoulder pain, fracture‐related pain, myofascial pain, and focal scar‐related neuropathy [11]. Alternative explanations were considered less likely clinically because the patient's symptoms were highly localized to the prior chest tube scar, were reproducibly provoked by palpation over the suspected entrapment sites, and were accompanied by winging most consistent with serratus anterior dysfunction. Electrodiagnostic studies were not performed before intervention.

*Musculoskeletal (MSK) ultrasonography (US)* was performed using an Alpinion XC90 Elite ultrasound system (ALPINION MEDICAL SYSTEMS Co. Ltd., Seoul, Republic of Korea) equipped with a high‐frequency linear transducer. Imaging parameters were standardized (depth, 3 cm; dynamic range, 60 dB), and a 7‐MHz setting was used to provide adequate penetration for visualization of the long thoracic nerve, the lateral cutaneous intercostal nerve branch, and the adjacent thoracic wall soft tissues. Coupling gel was applied to optimize acoustic transmission.

The LTN was identified by proximal‐to‐distal tracing along the superficial surface of the serratus anterior and distinguished from the more posterior thoracodorsal nerve based on its anatomical course and target muscle relationship [5,7]. On the symptomatic left side, the LTN demonstrated focal loss of fascicular definition, hypoechogenicity, and restricted excursion at the point where it pierced the axillary fascia over the sixth rib (Figure 3A; Video S1). Sonopalpation, including sonoguided digital palpation as previously described [12], reproduced the patient's concordant pain at this site.

**FIGURE 3:**
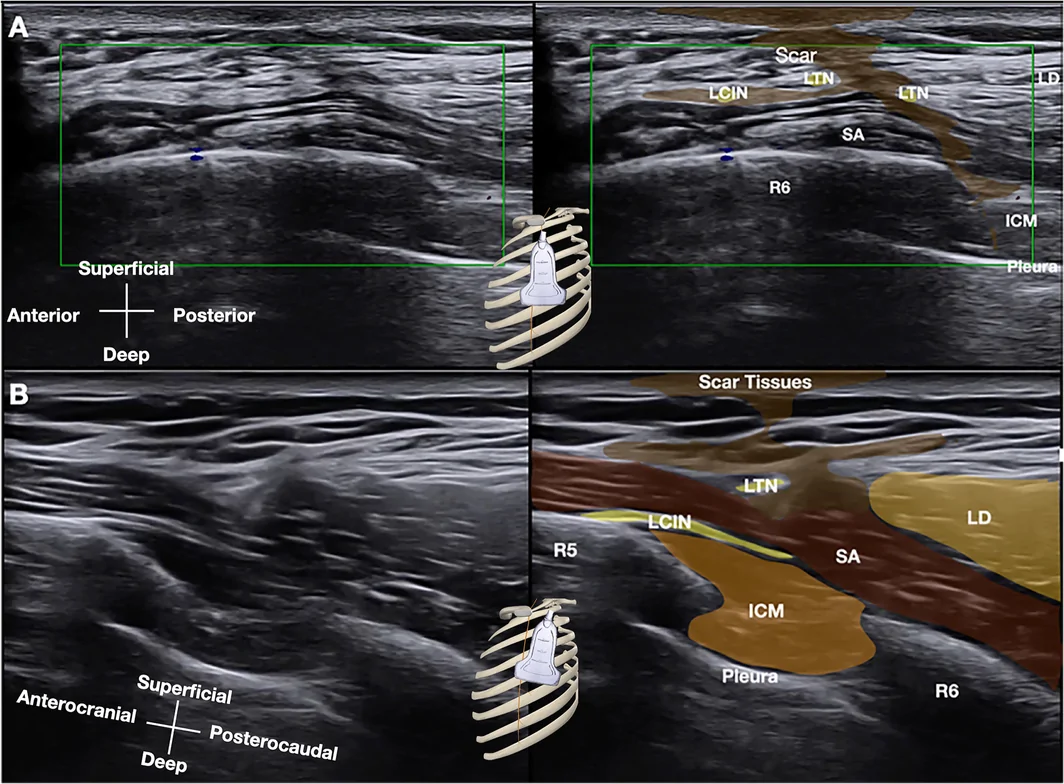
Ultrasound findings at the symptomatic left chest wall. (A) Transverse ultrasound image of the long thoracic nerve (LTN) at the level of the sixth rib as it pierces the axillary fascia. The nerve appears hypoechoic with loss of normal fascicular definition and reduced excursion relative to the contralateral side. (B) Transverse ultrasound image of the lateral cutaneous branch of the fifth intercostal nerve (LCIN) tethered within dense scar tissue at the prior chest tube insertion site. Orientation labels indicating anterior/posterior, cranial/caudal, and superficial/deep directions are provided.

In addition, the lateral cutaneous branch of the fifth intercostal nerve was identified near the midaxillary line where it traversed the lateral thoracic fascia and entered the scarred subcutaneous tissues. It appeared thickened and tethered within dense scar tissue at the chest tube insertion site (Figure 3B; Video S2). These abnormalities were not observed on the asymptomatic contralateral side (Video S3). Although a long‐axis view of the LCIN would have been desirable, this can be technically difficult to obtain reliably because of the nerve's small caliber and close proximity to the accompanying intercostal vessels.

### 2.3 Intervention

The patient was placed in the lateral decubitus position. Under sterile conditions and continuous ultrasound guidance, a 23‐gauge 6‐cm needle was advanced using a posterior‐to‐anterior in‐plane approach. Hydrodissection was performed using approximately 40 mL of 5% dextrose in water (D5W) in total, with 20 mL administered around each nerve to release the LTN from the constricting axillary fascia and the LCIN from the scar tether (Figure 4A,B; Videos S4, S5).

**FIGURE 4:**
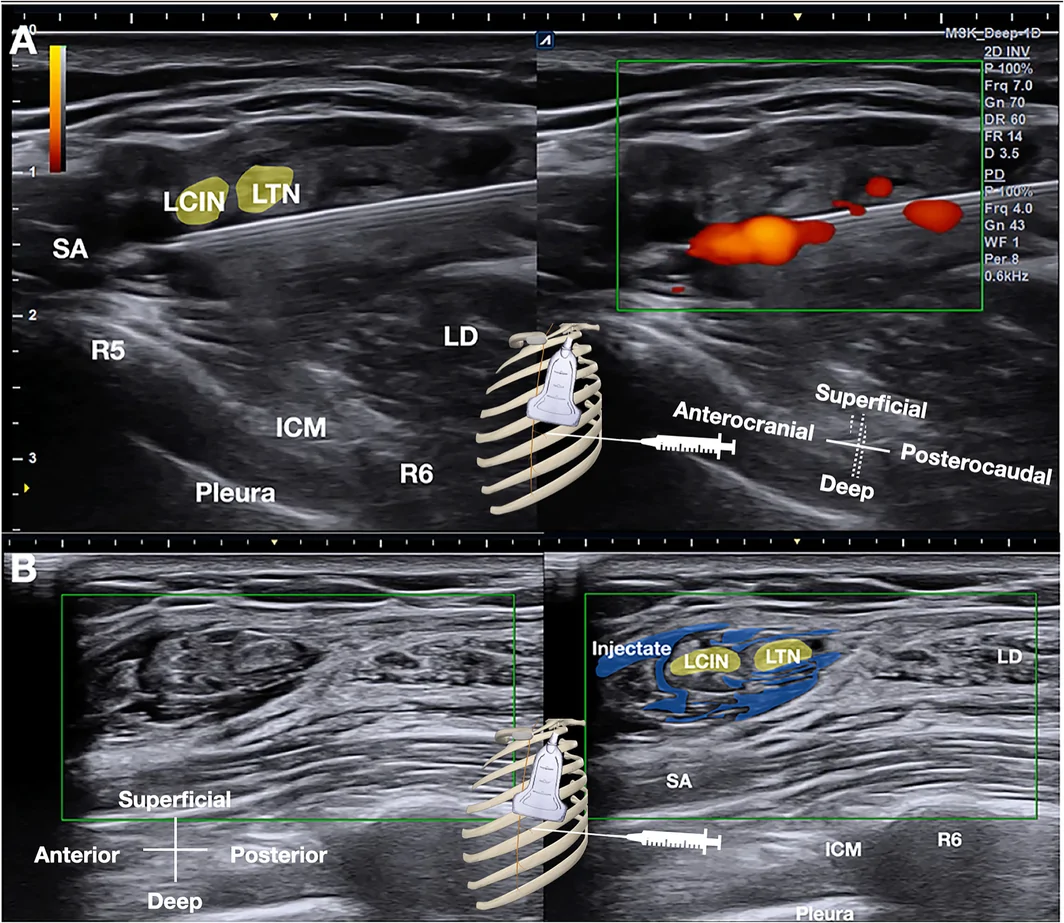
Ultrasound‐guided hydrodissection. (A) In‐plane posterior‐to‐anterior needle approach targeting the LTN and LCIN. (B) Successful fluid dispersion after hydrodissection, separating the nerve from surrounding fascia and scar tissue. Hydrodissection was performed using approximately 40 mL of 5% dextrose in water (D5W) in total, with 20 mL administered around each nerve. Orientation labels and scale markers are shown.

Immediately after the first procedure, the patient reported approximately 80% pain reduction, with improvement from NRS 9/10 to 2/10, and substantial reduction in pain during shoulder movement, including forward flexion. He subsequently underwent ultrasound‐guided hydrodissection at 2‐week intervals for a total of 5 treatment sessions over 10 weeks. Over the course of treatment, pain progressively improved, scapular winging diminished, and dysesthesia/allodynia resolved. Forward flexion remained full and was no longer accompanied by the previously aggravated pain. Manual muscle testing improved to 5/5 without pain. The SPADI improved during follow‐up from baseline values of 25 for pain and 15 for disability to 6 and 4, respectively. At the sixth follow‐up visit, pain and function were satisfactory, and the patient elected continued monitoring without further intervention. The appearance and discoloration of the chest tube scar also improved (Figure 5a–c). At 1‐year follow‐up, treatment efficacy remained sustained without recurrence of the previously reported symptoms.

**FIGURE 5:**
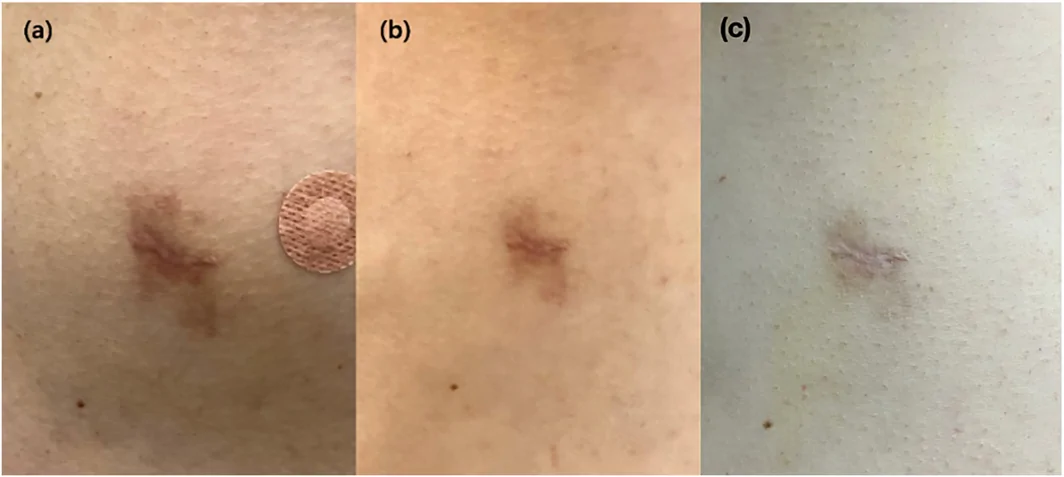
Clinical appearance of the chest tube scar before and after treatment. (a) Pretreatment hypertrophic scar with discoloration. (b) Scar appearance after serial hydrodissection treatments. (c) Scar appearance at 12 months, showing improvement in discoloration and skin texture.

Written informed consent for publication of this case report and accompanying images/videos was obtained from the patient. This report was prepared in accordance with the CARE reporting guidelines. The study was approved by the Institutional Review Board of Hallym University Kangnam Sacred Heart Hospital under expedited review (File No. HKS IRB2025‐08‐033; Doc No. HKS202508033‐HE002) and was conducted in accordance with the Declaration of Helsinki.

## 3 Discussion

This case highlights 2 clinically important points. First, persistent pain after chest tube thoracostomy may reflect not only intercostal nerve irritation but also concomitant involvement of the long thoracic nerve, producing a combined sensory and motor presentation. Second, musculoskeletal ultrasound can help localize the symptomatic lesion and guide targeted hydrodissection as a minimally invasive treatment.

Previous literature has described scapular winging after chest tube placement, most notably in the report by Hassan and Keaney [7], supporting the plausibility of chest tube‐related LTN injury. Anatomical work has further shown that the LTN frequently courses near the midaxillary line around the fifth and sixth rib/intercostal space region, placing it close to common thoracostomy trajectories [5]. By contrast, although intercostal nerve injury is recognized as a contributor to chronic post‐thoracic pain [8], concurrent focal entrapment of the LCIN together with the LTN after chest tube insertion appears to be rarely reported. We therefore describe this case conservatively as a rarely reported combined presentation, rather than overstate it as a definitively novel syndrome.

The clinical pattern in this case is anatomically coherent. The LTN supplies the serratus anterior and is essential for scapular protraction and stabilization; dysfunction classically produces medial scapular winging that becomes more apparent during forward pushing tasks [11]. The LCIN traverses the lateral thoracic wall near the midaxillary line and can generate localized pain, dysesthesia, and allodynia when irritated or trapped within scar tissue [8,9]. A chest tube passing through the lateral chest wall may injure either nerve directly or may create subsequent fibrosis and fascial tethering that leads to delayed entrapment.

The present case also underscores the value of careful interpretation of physical examination findings. Although punch‐out and push‐up maneuvers revealed obvious scapular winging, such tests are not specific for serratus anterior dysfunction in isolation [10]. Accordingly, our diagnosis was not based on bedside winging alone, but on the combination of focal neuropathic scar symptoms, ultrasound‐demonstrated focal nerve abnormalities, side‐to‐side asymmetry, and concordant symptom reproduction during sonopalpation.

An important interpretive issue is the absence of electrodiagnostic testing. Because electromyography and nerve conduction studies were not performed, we cannot definitively prove LTN axonopathy or exclude all alternative causes of scapular winging, including brachial plexopathy, cervical radiculopathy, or other periscapular neuropathies. This limitation is especially relevant because the patient improved immediately after hydrodissection. Most patients with true axonal LTN injury recover over months rather than minutes [11]. The rapid improvement observed here therefore suggests that dynamic fascial tethering, neurapraxia/conduction block, pain‐mediated motor inhibition, or a combination of these mechanisms contributed substantially to the clinical presentation. This interpretation is also consistent with the broader concept that scapular dyskinesis may be a nonspecific response to painful shoulder dysfunction rather than a marker of fixed denervation alone [13].

The ultrasound findings were clinically useful for several reasons. First, they permitted dynamic comparison with the contralateral side. Second, the LTN was identified by proximal‐to‐distal tracing, reducing the risk of misidentification with adjacent nerves. Third, sonopalpation reproduced the patient's symptoms at both suspected entrapment sites, strengthening the clinical relevance of the imaging findings. Fourth, ultrasound enabled a targeted intervention. Hydrodissection likely acted by mechanically separating the involved nerves from constricting fascia and scar tissue, restoring excursion, and reducing local mechanical irritation. D5W has been increasingly used for ultrasound‐guided nerve hydrodissection in entrapment neuropathies and may also have neuromodulatory effects, although the exact mechanism remains incompletely defined [9].

This case also has broader procedural implications. Although chest tube placement must often be performed urgently, awareness of vulnerable nerve anatomy may help reduce injury risk. Standard teaching emphasizes insertion within the triangle of safety and avoidance of the intercostal neurovascular bundle by advancing just above the rib [1, 2, 3]. However, cadaveric work has shown meaningful neurovascular variation in the fourth to sixth intercostal spaces at the midaxillary line, suggesting that the “safe zone” may be narrower than traditionally appreciated [14]. Additional anatomical work has further suggested that placement slightly anterior to the midaxillary line may reduce risk to the LTN and lateral cutaneous intercostal branches [5]. More broadly, thoracic procedures can endanger multiple neural structures, and familiarity with thoracic neural anatomy remains important when interpreting unexpected post‐procedural deficits [15].

Chest tube malposition has been reported to injure other neural structures, including the phrenic nerve and the ipsilateral sympathetic chain, resulting in diaphragmatic paralysis or Horner syndrome, respectively [16, 17]. Rarely, more distant neuropathic complications such as ulnar neuropathy have also been described in association with tube thoracostomy [18]. In selected non‐emergent or technically difficult cases, ultrasound may also assist procedural planning by clarifying pleural targets and adjacent structures [1].

An additional strength of this report is the longer follow‐up now available. The patient was monitored through 1 year after treatment, and the clinical benefit remained sustained without recurrence, supporting at least medium‐term durability of the observed response.

### 3.1 Limitations

This report has several limitations. First, it describes a single patient, which limits generalizability. Second, electrodiagnostic studies were not performed, so diagnostic certainty is limited and alternative explanations for scapular winging cannot be fully excluded. Third, although objective clinical outcome data were available, including preserved range of motion, manual muscle testing, and SPADI scores, formal goniometric measurements and serial ultrasound morphometric measurements were not obtained. Fourth, although follow‐up now extends to 1 year and demonstrated sustained benefit without recurrence, durability beyond this interval remains unknown and should be evaluated in larger prospective studies. Finally, although the temporal relationship between hydrodissection and symptom relief strongly supports treatment benefit, causality cannot be proven in a single uncontrolled case.

## 4 Conclusion

Persistent chest wall or shoulder pain after chest tube thoracostomy should prompt consideration of iatrogenic peripheral nerve injury or entrapment, particularly when accompanied by focal scar allodynia or scapular winging. This case suggests that combined involvement of the long thoracic nerve and a lateral cutaneous intercostal branch may occur and may be identifiable with focused neuromuscular examination and musculoskeletal ultrasound. Ultrasound‐guided hydrodissection may offer a useful minimally invasive treatment option in selected patients.

## Author Contributions

All authors have made substantial intellectual contributions to the manuscript. **Yonghyun Yoon:** conceptualization, design of the article, investigation, project administration, writing – original draft, writing – review and editing, supervision. **King Hei Stanley Lam:** conceptualization, design of the article, investigation, methodology, visualization, writing – original draft, writing – review and editing, supervision. **Daniel Chiung‐Jui Su:** project administration, writing – review and editing, validation. **Teinny Suryadi:** visualization, writing – review and editing, validation. **Anwar Suhaimi:** visualization, writing – review and editing, validation. All authors have read and approved the final submitted manuscript.

## Funding

The authors have nothing to report.

## Ethics Statement

This study was approved by the Institutional Review Board of Hallym University Kangnam Sacred Heart Hospital under expedited review (File No. HKS IRB2025‐08‐033; Doc No. HKS202508033‐HE002). Approval was granted on September 10, 2025, with notification issued on September 12, 2025.

## Consent

Written informed consent for publication of the case details and accompanying images/videos was obtained from the patient.

## Conflicts of Interest

The authors declare no conflicts of interest.

## Supporting information


**Video S1:** papr70195‐sup‐0001‐VideoS1.mp4. *Ultrasound evaluation of the long thoracic nerve*. Dynamic ultrasound tracing of the LTN on the symptomatic side, demonstrating focal hypoechogenicity, loss of fascicular definition, and restricted excursion where the nerve pierces the axillary fascia over the sixth rib. Symptom reproduction with sonopalpation is demonstrated.

**Video S2:** papr70195‐sup‐0002‐VideoS2.mp4. *Ultrasound evaluation of the lateral cutaneous intercostal nerve branch*. Dynamic ultrasound of the lateral cutaneous branch of the fifth intercostal nerve demonstrating focal tethering/thickening within scar tissue at the prior chest tube insertion site, with reproduction of concordant symptoms on sonopalpation.

**Video S3:** papr70195‐sup‐0003‐VideoS3.mp4. *Contralateral comparison*. Ultrasound examination of the asymptomatic contralateral side showing preserved fascicular appearance and mobility, without the focal abnormalities seen on the symptomatic side.

**Video S4:** papr70195‐sup‐0004‐VideoS4.mp4. *Ultrasound‐guided hydrodissection technique*. Real‐time in‐plane needle advancement in a posterior‐to‐anterior direction for hydrodissection of the LTN and LCIN.

**Video S5:** papr70195‐sup‐0005‐VideoS5.mp4. *Successful hydrodissection endpoint*. Visualization of fluid dispersion separating the involved nerves from surrounding fascia and scar tissue.

## Data Availability

Data sharing is not applicable to this article because no datasets were generated beyond the clinical materials presented in this case report.
